# Neural mechanisms underlying the interactive exchange of facial emotional expressions

**DOI:** 10.1093/scan/nsaf001

**Published:** 2025-01-17

**Authors:** Leon O H Kroczek, Andreas Mühlberger

**Affiliations:** Department of Psychology, Clinical Psychology and Psychotherapy, Regensburg University, Universitätsstraße 31, Regensburg 93053, Germany; Department of Psychology, Clinical Psychology and Psychotherapy, Regensburg University, Universitätsstraße 31, Regensburg 93053, Germany

**Keywords:** social interaction, EEG/ERP, virtual agents, emotion, facial expressions

## Abstract

Facial emotional expressions are crucial in face-to-face social interactions, and recent findings have highlighted their interactive nature. However, the underlying neural mechanisms remain unclear. This electroencephalography study investigated whether the interactive exchange of facial expressions modulates socio-emotional processing. Participants (*N* = 41) displayed a facial emotional expression (angry, neutral, or happy) toward a virtual agent, and the agent then responded with a further emotional expression (angry or happy) or remained neutral (control condition). We assessed subjective experience (valence, arousal), facial EMG (Zygomaticus, Corrugator), and event-related potentials (EPN, LPP) elicited by the agent’s response. Replicating previous findings, we found that an agent’s happy facial expression was experienced as more pleasant and elicited increased Zygomaticus activity when participants had initiated the interaction with a happy compared to an angry expression. At the neural level, angry expressions resulted in a greater LPP than happy expressions, but only when participants directed an angry or happy, but not a neutral, expression at the agent. These findings suggest that sending an emotional expression increases salience and enhances the processing of received emotional expressions, indicating that an interactive setting alters brain responses to social stimuli.

## Introduction

Facial emotional expressions are critical in social interactions, revealing a person’s affective state and intentions, and coordinating exchanges (Frith 2009, Lange et al. 2022). The emotion as social information model (Van Kleef 2009) suggests that perceiving an emotional expression (e.g. facial, body, or voice) impacts the observer in two ways. First, it can evoke an affective response either through mimicry (Dimberg 1982, Dimberg et al. 2000, Hess and Bourgeois 2010) or socio-emotional evaluation, such as liking someone who smiles or disliking an angry person (Salazar Kämpf et al. 2018). Second, it allows observers to infer the mental state, feelings, and intentions of the person displaying the expression (Van Kleef et al. 2011), thereby helping to identify the likely cause of the expression and inform subsequent behavior (Kroczek et al. 2021, 2024). For example, a smile from a stranger on a train might indicate affiliative intentions, prompting reciprocal behavior such as smiling back or greeting. Conversely, an angry expression might suggest that the person should be left alone. These examples illustrate how emotional expressions facilitate the coordination of interpersonal behavior in social contexts.

In real-time face-to-face interactions, emotional expressions emerge from dynamic exchanges between interactive partners (Heerey and Crossley 2013). During a dyadic exchange, individuals both send and receive emotional signals, interpreting them in the context of preceding expressions. This requires nested representations of others’ inferences (Lehmann et al. 2024), that is, one must consider the other person’s interpretations of one’s previous expressions. Although interactivity is a hallmark of human social behavior (Bolis et al. 2023), most studies have focused on noninteractive paradigms in which participants passively observe emotional expressions. Recent arguments suggest that studying social processes without real interaction may lead to reduced or different mechanisms compared to interactive situations (Fischer and van Kleef 2010, Risko et al. 2012, Redcay and Schilbach 2019). Real-time interactions, however, are challenging to investigate because of uncontrollable variables that may confound the processes of interest (Hadley et al. 2022). Therefore, virtual interactions with agents have been proposed as a solution that offers fine-grained control over verbal and nonverbal cues (Bombari et al. 2015). Overall, the study of social behavior should include genuinely interactive designs where a person’s actions elicit responses from others, and virtual social interactions can be beneficial for this purpose.

The interpersonal effects of facial emotional expressions are often studied through emotional mimicry, a mechanism in which observing a facial expression activates the corresponding facial muscles in the observer (Dimberg 1982). Emotional mimicry has been explained by the automatic matched-motor hypothesis, which posits a direct perception-action link (Chartrand Tanya and Bargh John 1999). Recent models, however, highlight the influence of communicative intentions and affiliative goals (Hess 2021), suggesting that social context modulates mimicry responses. Mimicry, for instance, is more consistent for positive emotional expressions in social interactions (Hess and Bourgeois 2010) and is enhanced by affiliative intentions (Salazar Kämpf et al. 2018). A recent study examined mimicry in an interactive context. Participants directed facial expressions (smile, neutral, and frown) at a virtual agent, which then responded with a smile or frown (Kroczek and Mühlberger 2022). Consistent with passive paradigms, observing a smile compared to a frown resulted in increased activation of M. zygomaticus. Crucially, this effect was most pronounced when participants initially smiled at the agent and disappeared when they first frowned. Similarly, initiating an interaction with a smile compared to a frown increased the pleasantness of an agent’s smile. This study indicated that the interactive context in face-to-face social interactions influences the evaluation of facial emotional expressions. However, the underlying neural mechanisms remain unknown.

Electroencephalography (EEG) enables the study of temporal dynamics in face processing, with event-related potentials (ERPs) linked to early (P1, N170), mid-latency (early posterior negativity, EPN), and late (late positive potential, LPP) processing stages (Wieser and Brosch 2012, Hinojosa et al. 2015, Schindler and Bublatzky 2020, Schindler et al. 2023). While emotional processing has been already linked to the N170 component (Hinojosa et al. 2015, Schindler et al. 2023), the EPN stage involves early attentional processing related to prioritized processing of emotional information and has been demonstrated for a range of emotional stimuli (e.g. faces, scenes, and words). Finally, the LPP concerns higher-order evaluation of emotional content, such as controlled attention or appraisal. The LPP is modulated by task relevance, i.e. effects of emotion were only observed when the task required attending to emotional information in the stimulus (Schindler et al. 2020, Krasowski et al. 2021). Furthermore, the LPP was found to be sensitive to personal relevance, for instance, when faces were introduced as future interaction partners or when an angry face stimulus was directly facing participants (Bublatzky et al. 2014, 2017). These findings suggest that emotional information in faces is processed early and adapts to task demands and personal relevance. This has implications for face-to-face social interactions, where a direct reciprocal exchange should increase relevance. An interactive paradigm is essential for testing this notion.

This study aimed to determine whether social interaction with a virtual agent influences the neural processing of facial emotional expressions following a previously established paradigm (Kroczek and Mühlberger 2022, 2023). Participants were instructed to display a facial emotional expression (happy, neutral, or angry) toward a virtual agent on a screen (hence called “initial expression”). The virtual agent then responded with another facial emotional expression (happy, neutral, or angry; hence called “response expression”). Continuous measurements of facial EMG and EEG were conducted to analyze mimicry and ERP responses to the agent’s facial expressions (EPN and LPP components). It was expected that the results would replicate the findings of Kroczek and Mühlberger (2022), demonstrating an interaction between the participant’s initial expression and the agent’s response expression in both valence ratings and Zygomaticus muscle activation. Additionally, it was hypothesized that the participant’s initial facial expression would influence the neural processing of the agent’s subsequent response expression. We expected this interaction effect for the LPP as an indicator of higher-order evaluative processing, given that sending a facial emotional expression to an interactive partner should directly influence the relevance of a response expression (Bublatzky et al. 2014, 2017). However, because relevance effects have been shown for both angry and happy facial expressions, we did not predict the direction of the interaction effect.

## Methods

### Participants

Forty-one healthy volunteers (34 females, mean age = 22.70 years, SD = 5.08, range = 18–40) participated in the study. They reported no neurological or mental disorders and had normal or corrected-to-normal vision. Four participants were excluded from the EMG and EEG analyses due to technical issues. The study adhered to the Declaration of Helsinki and was approved by the University of Regensburg’s ethics committee (19-1417-101). All participants provided written informed consent and received course credit for participation.

### Stimulus material

The stimulus material has been used in previous studies (Kroczek and Mühlberger 2022, 2023). The stimuli comprised video clips of four virtual agents (two females, two males) created with MakeHuman (v 1.1.1, http://www.makehumancommunity.org) and animated using Blender (v2.79, Blender Foundation). Agents were visualized as white, young adults (see Supplementary Fig. S1). Each video clip lasted 5500 ms, starting with the agents displaying a neutral facial expression. After 4000 ms, the facial expression changed to either angry or happy, or remained neutral. Emotional expressions transitioned within 500 ms and stayed visible for the remaining 1000 ms of the clip. In the neutral condition, the expression stayed the same throughout the clip. All videos featured agents with subtle head and body movements and eye blinks (5 different versions), resulting in 60 video clips (4 Agents × 3 Emotions × 5 Versions). Still frames of agents with happy, angry, or neutral expressions were rated for valence and arousal at the end of the experiment. Happy expressions were rated as most pleasant, followed by neutral and angry expressions. Both happy and angry expressions were rated more arousing than neutral ones, with angry expressions being slightly more arousing than happy ones. The emotional expression effect was consistent across the four agents (Supplementary Fig. S2).

### Procedure

After receiving instructions on the experimental procedures, participants completed questionnaires on demographic data (age, sex) and the Social Phobia Inventory (Connor et al. 2000). Subsequently, EEG electrodes were prepared, and participants were seated 60 cm away from a 21.5-inch LCD screen (HP EliteDisplay E221c, resolution: 1920 × 1080 pixels). The Social Phobia Inventory was not analyzed in the current study but data are available in the online repository.

**Figure 1.**
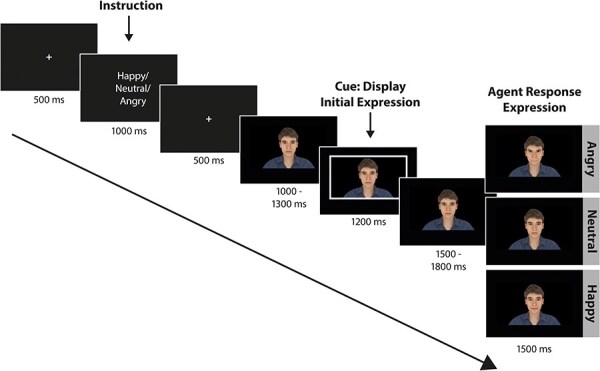
Experimental trial structure. The instruction informed participants about the facial emotional expression which they had to direct toward the virtual agent in the subsequent interaction. After the virtual agent appeared on the screen, a white rectangular frame around the virtual agent cued participants to display the facial emotional expression. After a jittered delay the virtual agent would then display a response facial emotional expression (angry or happy) or remain neutral.

Experimental procedures were controlled using PsychToolbox (Pelli 1997) in Matlab (v 8.6, MathWorks). In total, 360 pseudo-randomized trials were presented, with no more than three repetitions of initial expression, response expression, or virtual agent. Three practice trials were presented. All trials adhered to the same structure (Fig. 1), beginning with a fixation cross for 500 ms, followed by a 1000 ms instruction of the emotion to be directed at the virtual agent (Happiness, Anger, Neutral). After another 500 ms fixation cross, a virtual agent with neutral expression was presented. After a random interval between 1000 and 1300 ms, a white rectangular frame appeared around the agent, signaling participants to display the instructed emotion at the virtual agent using facial expressions (smile for happiness, frown for anger, no expression for neutral). The cue lasted for 1200 ms and participants were instructed to stop the facial emotional expression once the cue disappeared. After another delay between 1500 and 1800 ms, i.e. exactly 4000 ms after video onset, the agent displayed a facial response expression (Angry, Happy, Neutral) for 1500 ms until the clip ended. Subsequently, the next trial began, or, in the case of a catch trial, ratings were obtained. Catch trials comprised 20% of all trials (8 trials per condition). Participants rated arousal and valence regarding their interaction with the virtual agent on a 9-point Likert scale (“How high was your arousal with the previous person?”/“How pleasant did you feel with the previous person?”; 1 = very low arousal/very unpleasant, 9 = very high arousal/very pleasant). The entire experiment lasted approximately 45 min. There were self-determined breaks after every 80 trials.

### Prestudy: detection point of emotional expression

The study used dynamic video clips, in which agents displayed a neutral expression for 4000 ms before transitioning to an emotional expression over 500 ms. EEG analysis required precise onset timing for differentiating emotional expressions. Therefore, a pretest with an independent sample (*N* = 16) used a gating procedure (Pell et al. 2011, Kroczek et al. 2021) to determine when the transition from neutral to emotional expression was detectable. Participants viewed video clips with varying degrees of transition completeness, starting with a neutral expression for 3000 ms, followed by a transition to angry, happy, or remaining neutral. The transition duration varied from 1 to 20 frames. Participants identified the expressions as “angry,” “neutral,” or “happy” in a forced-choice paradigm after each clip. A total of 240 trials were presented in random order and the percentage of correctly identified expressions was measured for each number of presented transition frames (Supplementary Fig. S3). For each participant, the frame number at which the correct expression was detected ≥ 50% was identified. Results showed the mean detection point for emotional expressions was 7.41 frames (SD = 1.07). The detection point was therefore set to eight frames (133 ms at 60 Hz) for ERP and EMG analysis.

### EEG/EMG recording and preprocessing

EEG data were collected from 32 Ag/AgCl passive electrodes positioned according to the extended 10–20 system using an elastic cap (Easy Cap), with impedances kept below 5kΩ. Additional bipolar electrodes measured Zygomaticus and Corrugator muscle activity using two 6 mm Ag/AgCl electrodes per muscle, on the left side of the face following Fridlund and Cacioppo (1986) as well as vertical and horizontal eye movements. Data were recorded at 1000 Hz (NeurOne Tesla EEG/MRI System, Bittium), online-referenced to FCz, with AFz as ground.

Data preprocessing was conducted in Matlab (v 9.7.0) using EEGLAB (v2022.0, Oostenveld et al. 2011). EMG data preprocessing included applying a bandpass filter (30–500 Hz) and a 50 Hz notch filter (−6 dB, half amplitude, noncausal, zero-phase, Hamming window). Data were rectified, integrated (moving average of 125 ms window), log-transformed to minimize skewness, and segmented into 2000 ms epochs around the change detection point, with 500 ms prestimulus and 1500 ms poststimulus periods. Baseline correction was applied. Trials were excluded if participants did not display the instructed facial emotional expression (M = 22.57, SD = 21.54). To identify these trials, a pre-established procedure was used (Kroczek and Mühlberger 2023), comparing EMG activity postcue onset against a muscle-specific threshold. For instructed smiles or frowns, Zygomaticus or Corrugator activity had to exceed a muscle-specific threshold. For a neutral expression, neither Zygomaticus nor Corrugator activity could exceed the thresholds. The threshold was set at 25% of the 90th percentile of activation maxima in all trials with a specific facial emotional instruction.

EEG data preprocessing included artifact correction with independent component analysis (ICA). Therefore, a two-step procedure was conducted. First, data were preprocessed to optimize ICA decomposition. Here, raw EEG was filtered with a 1 Hz high-pass filter (−6 dB, half amplitude, noncausal zerophase, Hamming window), re-referenced to the average, and ICA was performed using runica with the infomax algorithm (Delorme and Makeig 2004). In a second step, the ICA components were then projected onto another dataset with different filter settings, which was then used for the actual ERP analysis. The second dataset was created by filtering the raw EEG with a 0.1–30 Hz bandpass filter (same filter specification as above) and re-referencing to the average. ICA components from the first step were projected onto this new dataset, and components associated with eye-blinks and eye-movements were removed. Data were segmented into epochs [−200 to 1000 ms] time-locked to the change detection point of the virtual agent’s facial emotional expression. For trials with a neutral expression, the same timepoint was defined as the onset. Epochs exceeding ± 80 µV were rejected (mean trials rejected = 10.82%, SD = 15.69%). A prestimulus interval of 200 ms was used for baseline correction, and trials in which participants displayed the incorrect facial emotional expression (see above) were excluded.

Note that participants’ display of the initial facial emotional expressions was linked to strong EMG activation (Supplementary Fig. S4), which took several seconds to return to baseline. To control for this residual muscle activation in the EMG and ERP analyses, we calculated difference waves by subtracting the neutral response from both angry and happy responses for each initial expression condition. Using this subtraction allowed us to remove the residual muscle activation from the participant’s own expression while preserving the activation which was elicited by the agent’s response expression. This procedure was conducted for both the EMG and ERP data. Supplementary Figs S6 and S7 show ERP data before subtracting in all three response conditions (angry, happy, neutral).

### Statistical analyses

Repeated measures analyses of variance (ANOVAs) were used to analyze ratings (factors: Initial Expression, Response Expression) and EMG data (factors: Initial Expression, Response Expression, Time Window; 15 consecutive 100 ms windows). Significant Time Window interactions were further analyzed by averaging data across windows. The EPN (200–350 ms; channels PO9, PO10, P7, P8, O1, Oz, O2; Herbert et al. 2013, Bagherzadeh‐Azbari et al. 2023) and LPP (400–800 ms; channels CP1, CP2, Pz, P3, P4) components were analyzed using repeated measures ANOVAs (factors: Initial Expression, Response Expression; Schindler et al. 2020, Krasowski et al. 2021). Greenhouse–Geisser adjustments corrected for sphericity violations, and *post-hoc t*-tests with Holm correction followed significant interactions (Greenhouse and Geisser 1959; Holm 1979). Note that for EMG and ERP analyses, the levels angry and happy of the factor Response Expression included the difference between angry-neutral response expression conditions and happy-neutral response expression conditions for each initial expression to control for confounding muscle activation elicited by participants’ active display of an emotional facial expression (see above). Exploratory correlations examined associations between ratings, EMG, and ERP responses. Rating data were recalculated as emotional-neutral differences for each initial expression level. Zygomaticus (0–1500 ms) and Corrugator (200–500 ms) activation were averaged based on main analysis results. Alpha was set at 0.05. Stimulus materials, experimental scripts, analysis scripts, and anonymized rating data are available online (https://osf.io/ghdrm/). Raw EEG data are available upon request.

## Results

### Ratings

**Figure 2.**
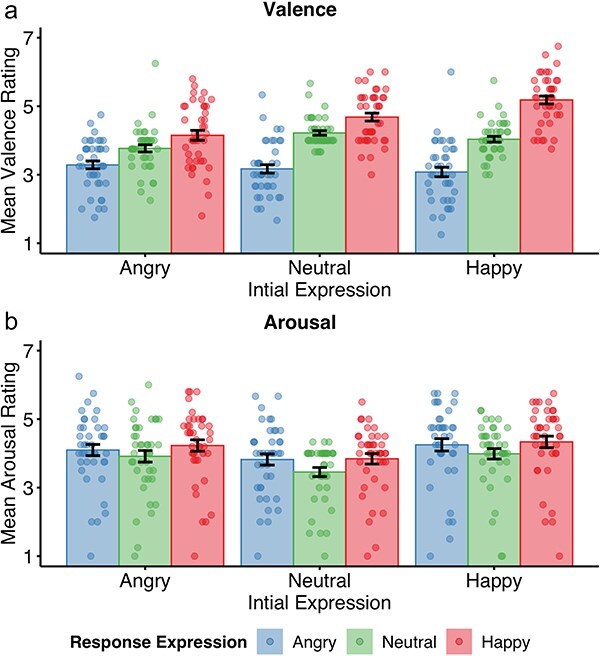
Valence and arousal ratings as a function of the initial facial emotional expression by the participant (angry, neutral, happy) and the facial emotional response expression returned by the virtual agent (angry, neutral, happy).

Analysis of valence ratings (Fig. 2a) revealed an interaction between Initial Expression and Response Expression, F(4160) = 16.67, *P* < .001, η_p_^2^ = 0.29 (ε = 0.60), a main effect of Initial Expression, F(2,80) = 16.58, *P* < .001, η_p_^2^ = 0.29 (ε = 0.73), and a main effect of Response Expression, F(2,80) = 67.74, *P* < .001, η_p_^2^ = 0.63 (ε = 0.57). An agent’s happy expression was most pleasant following an initial happy, intermediate pleasant following an initial neutral, and least pleasant following an angry expression [happy-neutral: t(40) = 4.86, *P* < .001, d = 0.76, neutral-angry: t(40) = 4.75, *P* = .001, d = 0.74]. Whereas an agent’s neutral expression was most pleasant following an initial neutral compared to an initial angry, t(40) = 4.70, *P* <.001, d = 0.73, or initial happy expression, t(40) = 2.19, *P* = .045, d = 0.34. Valence of angry response expression did not differ between initial expressions (all *P*s >.10).

Analysis of arousal ratings (Fig. 2b) revealed a main effect of Initial Expression, F(2,80) = 15.57, *P* < .001, η_p_^2^ = 0.28 (ε = 0.83), and a main effect of Response Expression, F(2,80 = 12.32, *P* < .001, η_p_^2^ = 0.24, but no interaction between Initial Expression and Response Expression, F(4, 160) = 0.47, *P* = .761, η_p_^2^ = 0.01. Specifically smiling and frowning increased arousal compared to displaying a neutral expression [happy-neutral: t(40) = 4.55, *P* <.001, d = 0.71; angry-neutral: t(40) = 3.99, *P* < .001, d = 0.62], but there was no difference between smiling and frowning, t(40) = 1.43, *P* = .162, d = 0.22. Similarly, arousal ratings were higher for happy and angry compared to neutral response expressions [happy-neutral: t(40) = 4.86, *P* < .001, d = 0.75; angry-neutral: t(40) = 3.59, *P* = .002, d = 0.56] but there was no difference between happy and angry response expressions, t(40) = 0.85, *P* = .399, d = 0.13.

Overall, sending a facial emotional expression toward an interactive partner modulates pleasantness of a response smile, while arousal is not influenced by the interplay of sending and receiving an emotional expression.

### EMG response

#### M. Zygomaticus

**Figure 3.**
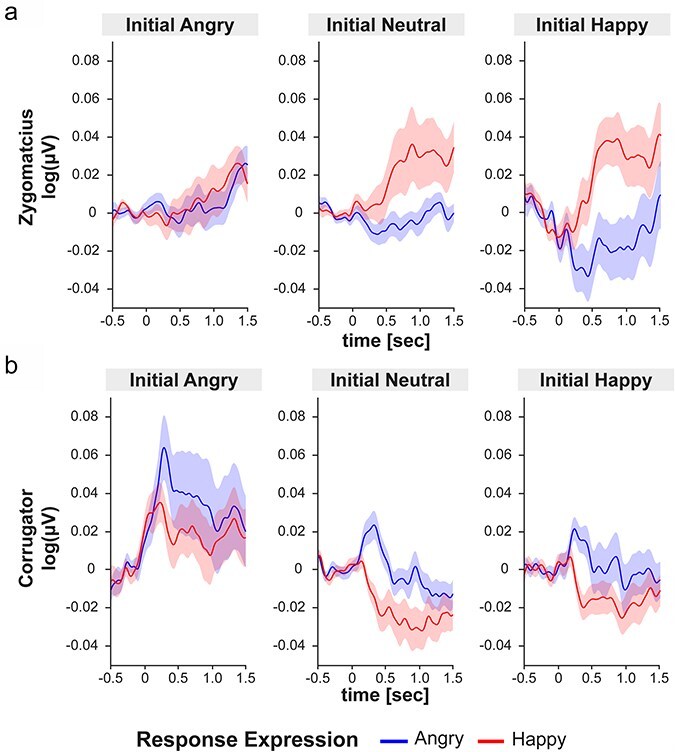
EMG activation in the Zygomaticus (a) and Corrugator muscle (b) following happy or angry response expressions of the virtual agents as a function of the initial emotional expression that was displayed by the participant. Shaded areas represent the standard error of the mean.

Analysis of Zygomaticus activity showed a main effect of Response Expression, F(1,36) = 6.77, *P* = .013, η_p_^2^ = 0.16, a main effect of Time Window, F(14 504) = 6.78, *P* = .002, η_p_^2^ = 0.16 (ε = 0.15), and interactions between Response Expression and Time Window, F(14, 504) = 4.79, *P* = .017, η_p_^2^ = 0.12 (ε = 0.12), and Initial Expression and Response Expression, F(2,72) = 4.90, *P* = .010, η_p_^2^ = 0.12. Happy response expressions triggered stronger Zygomaticus activation than angry expressions, particularly within 200 to 1400 ms post onset of the agent’s response expression. Furthermore, stronger Zygomaticus activation was elicited for happy versus angry response expressions when participants initially displayed a happy expression, t(36) = 3.48, *P* = .004, d = 0.57, but not for initially neutral, t(36) = 1.99, *P* = .109, d = 0.33, or angry expressions, t(36) = 0.20, *P* = .840, d = 0.03 (see Fig. 3a). Results indicate that Zygomaticus mimicry effects are amplified when a smile is directed at an interactive partner.

#### M. Corrugator

Analysis of Corrugator activity (Fig. 3b) revealed a main effect of Initial Expression, F(2,72) = 6.53, *P* = .008, η_p_^2^ = 0.15 (ε = 0.66), and Time Window, F(14 504) = 4.93, *P* = .003, η_p_^2^ = 0.12 (ε = 0.21), and an interaction effect between Response Expression and Time Window, F(14 504) = 4.92, *P* = .004, η_p_^2^ = 0.16 (ε = 0.19). Angry response expression elicited higher Corrugator activation than happy ones in a time window of 200 to 500 ms, t(36) = 3.62, *P* < .001, d = 0.59. Corrugator activity was also higher when participants had initially displayed an angry compared to a neutral, t(36) = 3.12, *P* = .011, d = 0.51, or happy expression (marginal significant), t(36) = 2.32, *P* = .053, d = 0.38. These data demonstrate a mimicry effect for angry expressions in the Corrugator which was not modulated by sending an initial facial emotional expression.

### Event-related potentials

#### EPN

**Figure 4.**
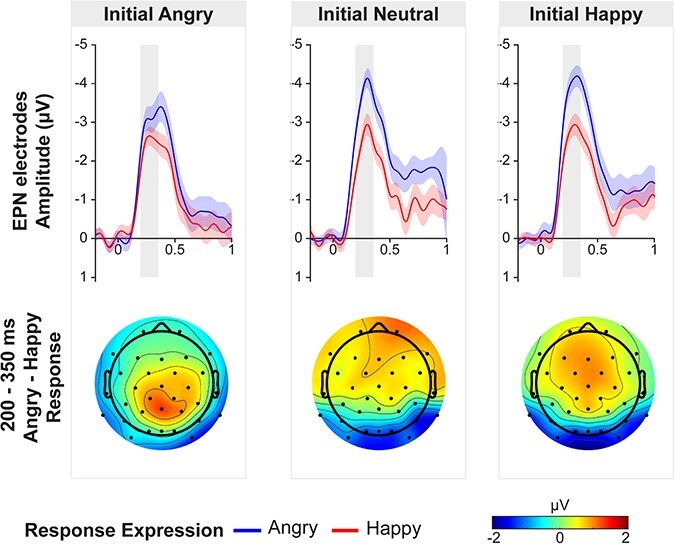
Top: ERPs elicited by the agent’s facial expression as a function of the initial emotional expression of the participant (left = angry, middle = neutral, right = happy) and the response expression of the agent (angry = blue line, happy = red line) averaged across temporo-occipital electrodes (PO7, PO8, P7, P8, O1, Oz, O2). ERPs are time-locked to the detection point of the facial emotional expression. Single ERPs always reflect the difference from the neutral control condition (no agent response) for the same initial expression. The 200–350 ms window in which EPN was analyzed is highlighted in grey. Shaded areas represent the standard error of the mean. Bottom: Topographical distribution of the emotion effect for each initial expression by subtracting ERPs to angry and happy response expression in a time window of 200-300 ms.

Analysis of the EPN investigated the effects of Initial Expression and Response Expression on temporo-occipital electrodes between 200 and 350 ms post detection point of the emotional response expression (Fig. 4). A repeated-measures ANOVA revealed a main effect of Response Expression, F(1,36) = 41.92, *P* < .001, η_p_^2^ = 0.54. Angry response expressions (M = −3.56, SD = 1.50) elicited a more negative ERP than happy response expressions (M = −2.60, SD = 1.04). There was no main effect of Initial expression, F(2,72) = 2.18, *P* = .121, η_p_^2^ = 0.06, and no interaction between Initial Expression and Response Expression F(2,72) = 2.68, *P* = .075, η_p_^2^ = 0.07.

#### LPP

**Figure 5.**
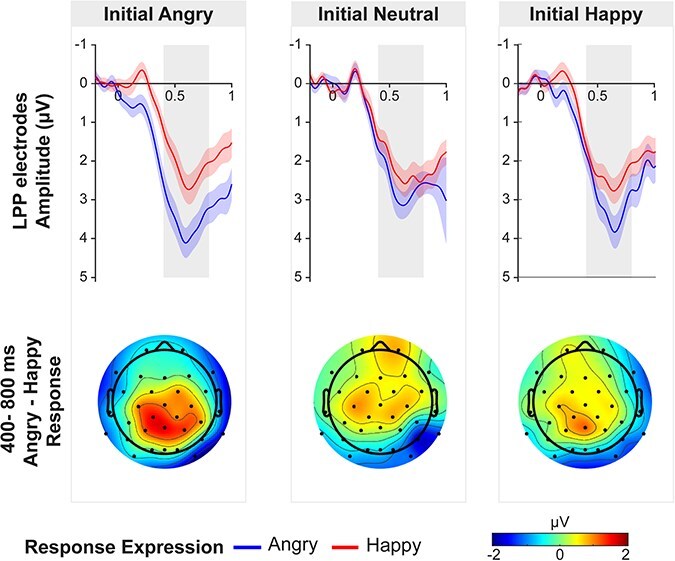
Top: ERPs elicited by the agent’s facial expression as a function of the initial emotional expression of the participant (left = angry, middle = neutral, right = happy) and the response expression of the agent (angry = blue line, happy = red line) averaged across centro-parietal electrode (CP1, CP2, Pz, P3, P4). ERPs are time-locked to the detection point of the facial emotional expressions. Single ERPs always reflect the difference from the neutral control condition (no agent response) for the same initial expression. The 400–800 ms window in which LPP was analyzed is highlighted in grey. Shaded areas represent the standard error of the mean. Bottom: Topographical distribution of the emotion effect for each initial expression by subtracting ERPs to angry and happy response expression in a time window of 400–800 ms.

The LPP component was examined in centro-parietal electrodes between 400 and 800 ms post detection point of the emotional response expression (Fig. 5). ANOVA results showed a main effect of Response Expression, F(1,36) = 26.55, *P* < .001, η_p_^2^ = 0.42, and an interaction between Initial Expression and Response Expression, F(2,72) = 3.83, *P* = .026, η_p_^2^ = 0.10. No main effect of Initial Expression was found, F(2,72) = 1.42, *P* = .249, η_p_^2^ = 0.04. *Post-hoc t*-tests revealed a more positive LPP for angry compared to happy response expressions following both angry, t(36) = 4.13, *P* < .001, d = 0.68, and happy, t(36) = 3.70, *P* = .001, d = 0.61, but not neutral initial expressions, t(36) = 1.90, *P* = .066, d = 0.32. The difference between angry and happy response expressions was larger after an angry initial expression compared to a neutral one, t(36) = 2.66, *P* = .035, d = 0.44, but no difference was found between angry versus happy initial expressions, t(36) = 1.82, *P* = .153, d = 0.29, or happy versus neutral initial expressions, t(36) = 0.92, *P* = .036, d = 0.15. Therefore, displaying an emotional facial expression at an interactive partner affected the LPP response to emotional expressions. A greater LPP for angry compared to happy expressions was only observed for initial angry and initial happy but not initial neutral conditions.

### Correlations

Pearson correlations investigated the relation of ratings, EMG, and ERP responses. After multiple comparisons correction (Holm), only two significant correlations remained (Supplementary Table S1): Zygomaticus activity correlated positively with valence ratings, r(220) = 0.25, *P* = .005, while Corrugator activity correlated positively with LPP amplitude, r(220) = 0.21, *P* = .036. In summary, the more pleasant an interaction was experienced, the more activation was elicited in the Zygomaticus muscle, and the more activation was elicited in the Corrugator muscle, the greater the LPP amplitude.

## Discussion

This study examined whether sending a facial emotional expression affects the processing and evaluation of response facial emotional expressions. Results showed two patterns: Firstly, smiling, as opposed to frowning, at an interactive partner increased pleasantness and mimicry of the partner’s smiles, replicating findings by Kroczek and Mühlberger (2022). Secondly, neurophysiological data revealed enhanced processing of the partner’s facial expressions when participants initially frowned or smiled but not when they displayed a neutral expression. Thus, while all measures were sensitive to the interplay of sending and receiving facial expressions, behavioral (ratings and mimicry) and neurophysiological measures appear to reflect different socio-affective processing mechanisms. Mimicry and ratings align with an affiliative mechanism, enhancing the evaluation and mimicry of positive expressions, whereas LPP data suggest a relevance mechanism, where facial expressions gain relevance following one’s own emotional expression.

LPPs elicited by an agent’s emotional expressions varied based on preceding facial expressions of the participant. Consistent with prior research, angry expressions induced a greater LPP than happy expressions (Blechert et al. 2012), which has been linked to enhanced processing of motivationally salient emotional information (Hajcak et al. 2009). This pattern emerged only when participants initially displayed angry or happy expressions, not neutral ones. Thus, the initial facial expression impacted the in-depth analysis of subsequent emotional expressions, likely due to increased (self-) relevance from the reciprocal nature of social interaction. This aligns with findings that LPP modulation is affected by perceived personal relevance (Bublatzky et al. 2017) or task demands, such as evaluating an expression’s emotionality (Schindler et al. 2020). These results suggest an adaptive neural mechanism for processing social signals with heightened personal relevance during face-to-face interactions. Additionally, negative emotional expressions may enhance processing due to their indication of significant social consequences.

A main effect of emotional expression was observed in the EPN component and an additional cluster analysis found that happy and angry expressions differed from 100 ms post detection point (Supplementary Material). This aligns with prior findings on early processing of emotional expressions in components like the N170 and EPN (Hinojosa et al. 2015, Schindler and Bublatzky 2020). Please note, however, that the nature of the present paradigm, i.e. display of the face stimulus preceding the onset of the facial emotional expression, precluded us from identifying clear P1 and N170 components in the ERP waveforms. Thus, the early effect observed here may not represent the initial processing of facial emotional expressions in components such as the P1 or N170 (which were reliably elicited when the virtual agent appeared, see Supplementary Fig. S5). Nonetheless, the increased negativity to angry expressions indicates early-stage processing of emotional information in the presented faces. Importantly, this processing stage did not vary based on the emotional expression participants initially sent to the agent, suggesting that early structural and attentional processing of emotional information is not influenced by preceding social interactive context.

Contrary to ERP findings, EMG responses and valence ratings show a different interaction pattern between sending and receiving facial emotional expressions. Increased Zygomaticus mimicry and more positive evaluations of happy response expressions following initial smiles compared to frowns suggest an affiliative function of reciprocal facial expressions, where sending a smile enhances the rewarding value of a returned smile, thereby boosting the mimicry response (Sims et al. 2012). This aligns with the mimicry in social context model (Hess 2021), which suggests that mimicry occurs only when sender and receiver have affiliative intentions. Our data support this model; sending a smile, communicating affiliative intent (Jack and Schyns 2015), increased mimicry responses compared to sending an angry expression, which communicates nonaffiliative intent. This interplay of mimicking and being mimicked may establish social bonds between partners (Wicher et al. 2023). Furthermore, although we observed a general mimicry effect for nonaffiliative (angry) expressions, it was not influenced by the initial emotional expression. These findings imply that affiliative intentions enhance mimicry effects for affiliative but not nonaffiliative expressions, supporting a socio-affective mechanism for the reciprocal exchange of facial emotional expressions in social interactions.

Few studies have simultaneously measured EEG and facial EMG. Achaibou et al. (2008) found a link between the amplitude of the P1 and N170 components and the strength of the EMG mimicry response. Another study suggested that later face processing stages, such as the P3, also vary with mimicry (Kuang et al. 2021). Contrary, the present study found no associations between mimicry and early stages of facial emotion processing. Keep in mind, however, that our paradigm did not isolate early components like the P1 or N170. Instead, we found that Corrugator muscle activation correlated with LPP amplitude, possibly indicating that enhanced processing of motivationally salient emotional information is associated with Corrugator activation, consistent with Grèzes et al. (2013), who found enhanced Corrugator activation for self-relevant processing. Furthermore, while no correlations were observed between Zygomaticus muscle activation and neural processes, Zygomaticus activation correlated with the evaluation of pleasantness, aligning with Sato et al. (2013). Again, this pattern suggests an affiliative mechanism enhancing mimicry and evaluative responses for affiliative signals on the one hand, and enhanced processing of emotionally salient and self-relevant information on the other hand.

While the present study is the first to investigate neural processes regarding the interactive exchange of facial emotional expressions, there are some limitations that need to be discussed. First, rather than real interactions, a “pseudo-interactive” hard-coded paradigm was used where participants were prompted to direct predetermined facial expressions at a virtual agent, who then responded after a random delay. This setup, while ensuring high experimental control, differs from real-life face-to-face interactions. For example, being instructed to display specific expressions may have reduced communicative intentionality and the feeling of social agency (Brandi et al. 2020). Additionally, the 1500–2000 ms delay is longer than typical face-to-face interaction delays (Heerey and Crossley 2013). However, these delays were still linked to a relatively high experience of responsiveness in a previous study (Kroczek and Mühlberger 2023). While these limitations need to be considered, it should be noted that sending a facial expression affected processing of subsequent expressions despite being only “pseudo-interactive”, suggesting that one’s actions create a meaningful social communicative context. Another limitation of this study is that early ERP components like the N170 elicited by facial emotional expressions could not be clearly identified, as we did not use static images or dynamic expressions starting at timepoint zero. Instead, we used naturalistic video stimuli where the agents appeared on the screen for several seconds before displaying emotional expressions. This approach was necessary for our experimental design and better reflects real-time social behavior. Note, however that we measured a typical N170 at video onset (see Supplementary Fig. S5). Future studies could therefore isolate specific early face processing components by introducing an empty screen before the agent’s response expression, allowing for clear onset measurement.

In summary, active engagement in face-to-face social interaction influences the neural and behavioral processing of facial emotional expressions. The results suggest that sending an emotional expression enhances higher-order evaluative processing of the response expression, while sending an affiliative expression boosts affiliative mimicry and pleasantness evaluation. These findings highlight that social interactive processes may trigger distinct mechanisms essential for navigating complex social environments.

## Supplementary Material

nsaf001_Supp
