# Supplementary material for: Neural mechanisms underlying the interactive exchange of facial emotional expressions
Source: Soc Cogn Affect Neurosci. 2025 Jan 17;20(1):nsaf001. doi: 10.1093/scan/nsaf001 (PMC11781275; doi:10.1093/scan/nsaf001)
Supplement: nsaf001_Supp [file nsaf001_supp.zip › scan-24-234-File002.pdf]

Supplementary Material: Neural mechanisms underlying the interactive exchange of facial emotional expressions

Leon O.H. Kroczek*[1] & Andreas Mühlberger[1]

[1] Department of Psychology, Clinical Psychology and Psychotherapy, Regensburg University

Ratings of facial emotional expressions for all virtual agents

To investigate whether there were differences in the evaluation of facial expressions between virtual agents, participants were asked to rate valence and arousal for still frames of each agent displaying either an angry, neutral or happy expression (Figure S1).

A repeated measures ANOVA of valence ratings revealed a main effect of *Emotion*, $F(2,80) = 135.22$, $p < .001$, $\eta_p^2 = 0.77$ ($\varepsilon = 0.66$), and a main effect of *Agent*, $F(3,120) = 4.26$, $p = .012$, $\eta_p^2 = 0.10$ ($\varepsilon = 0.79$), but no interaction between *Agent* and *Emotion*, $F(6,240) = 1.32$, $p = .264$, $\eta_p^2 = 0.03$ ($\varepsilon = 0.70$). Happy expressions were rated as more pleasant than neutral, $t(40) = 8.12$, $p < .001$, $d = 1.27$, and angry expressions, $t(40) = 13.05$, $p < .001$, $d = 2.04$. And neutral expressions were rated as more pleasant than angry expressions, $t(40) = 13.81$, $p < .001$, $d = 2.16$. With respect to agent identity, agent "Female 1" was rated as more pleasant than agent "Male 2", $t(40) = 4.10$, $p = .001$, $d = 0.64$. There were no other significant differences between agents with respect to valence ratings (all $p > .05$).

Similarly, analysis of arousal ratings revealed a main effect of *Emotion*, $F(2,80) = 19.06$, $p < .001$, $\eta_p^2 = 0.32$, and a main effect of *Agent*, $F(3,120) = 2.79$, $p = .044$, $\eta_p^2 = 0.07$, but no interaction between *Agent* and *Emotion*, $F(6,240) = 0.53$, $p = .788$, $\eta_p^2 = 0.01$ ($\varepsilon = 0.76$). Angry expressions were arousing than neutral, $t(40) = 6.10$, $p < .001$, $d = 0.95$, and happy expressions, $t(40) = 2.40$, $p = .021$, $d = 0.37$, and happy expressions were more arousing than neutral expressions, $t(40) = 3.92$, $p < .001$, $d = 0.61$. Post-hoc t-tests did not reveal any significant differences between agents with respect to arousal ratings (all $p > .05$ after correction for multiple comparisons).

Overall, the stimulus material was evaluated as intended and there were no differences in how facial emotional expressions were evaluated between agents.

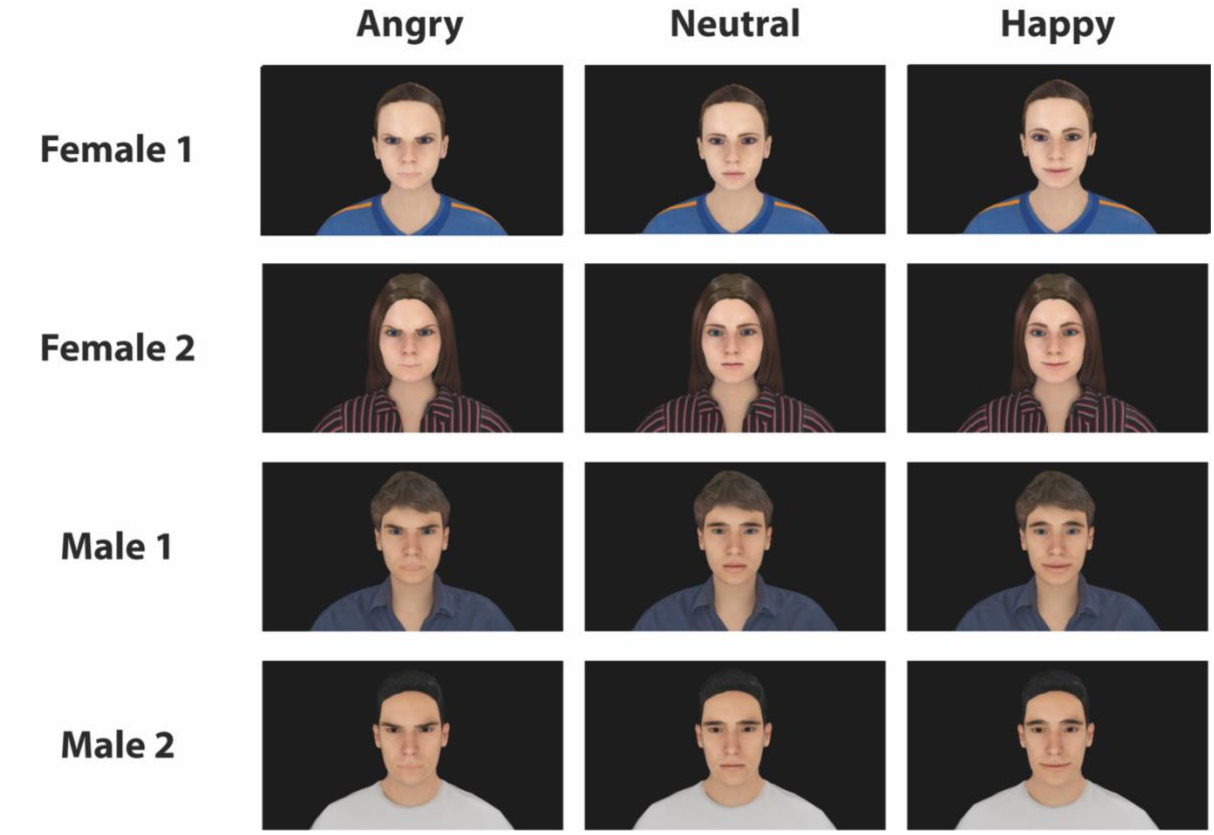

Figure S1: Still frames of the four virtual agents displaying an angry (left), neutral(middle), and happy facial expression (right).

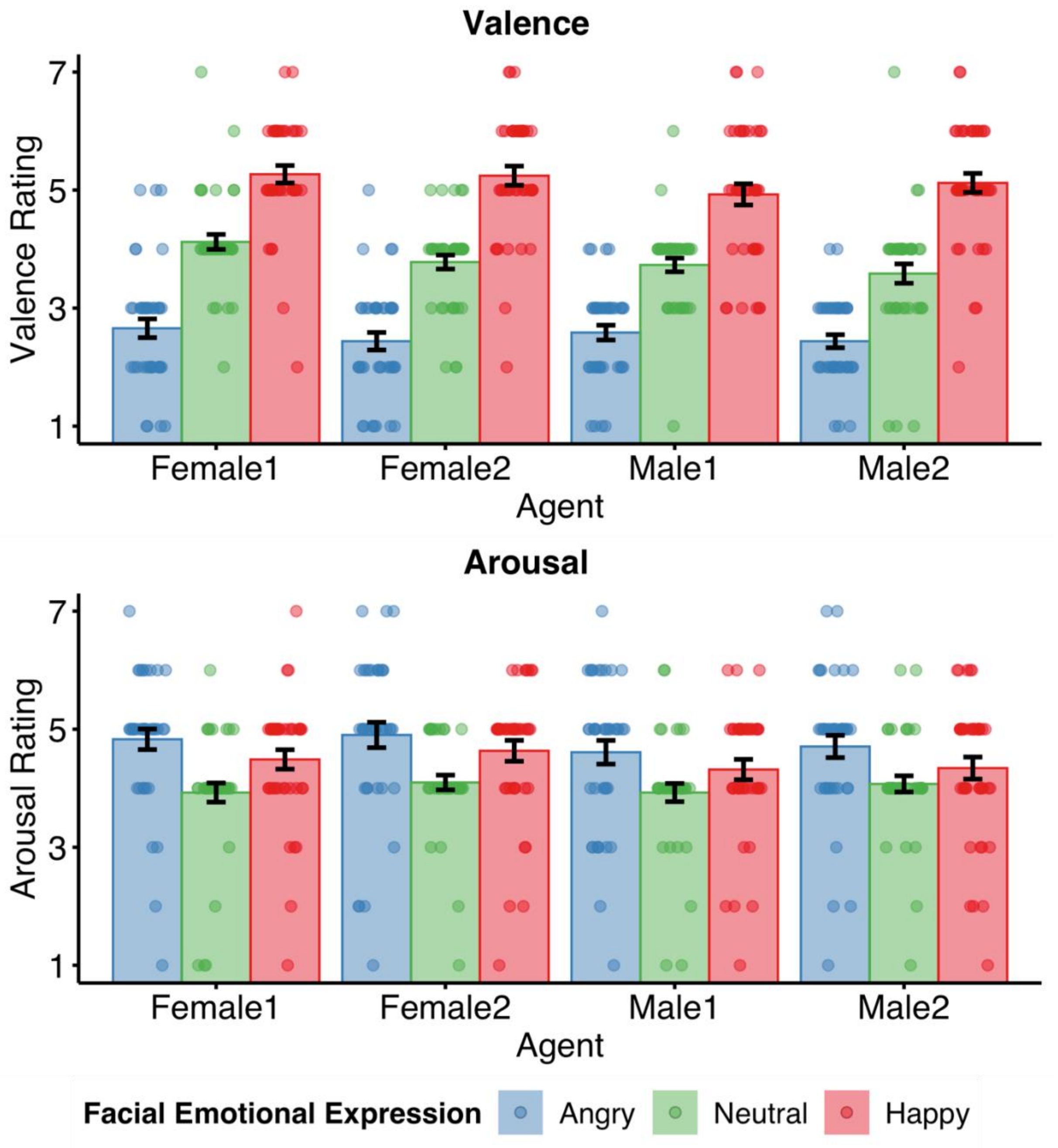

Figure S2: Valence (top) and Arousal ratings (bottom) as a function of facial emotional expression (angry, neutral, happy) and virtual agent. Error bars reflect standard error of the mean.

Detection accuracy as a number of frames in the pre-test

In order to test at which point in time a change from neutral to an emotional expression can be detected in the dynamic video clips a pre-test was conducted in which participants had to indicate the emotional expression which they observed in a video clip. The emotional expression transitioned from neutral to angry, from neutral to happy or remained neutral. The degree of completeness of the transition was manipulated for each video clip (1-20 frames of the transition were displayed). The detection point was calculated as the timepoint in which angry or happy expressions could be detected with a proportion of >= 0.5.

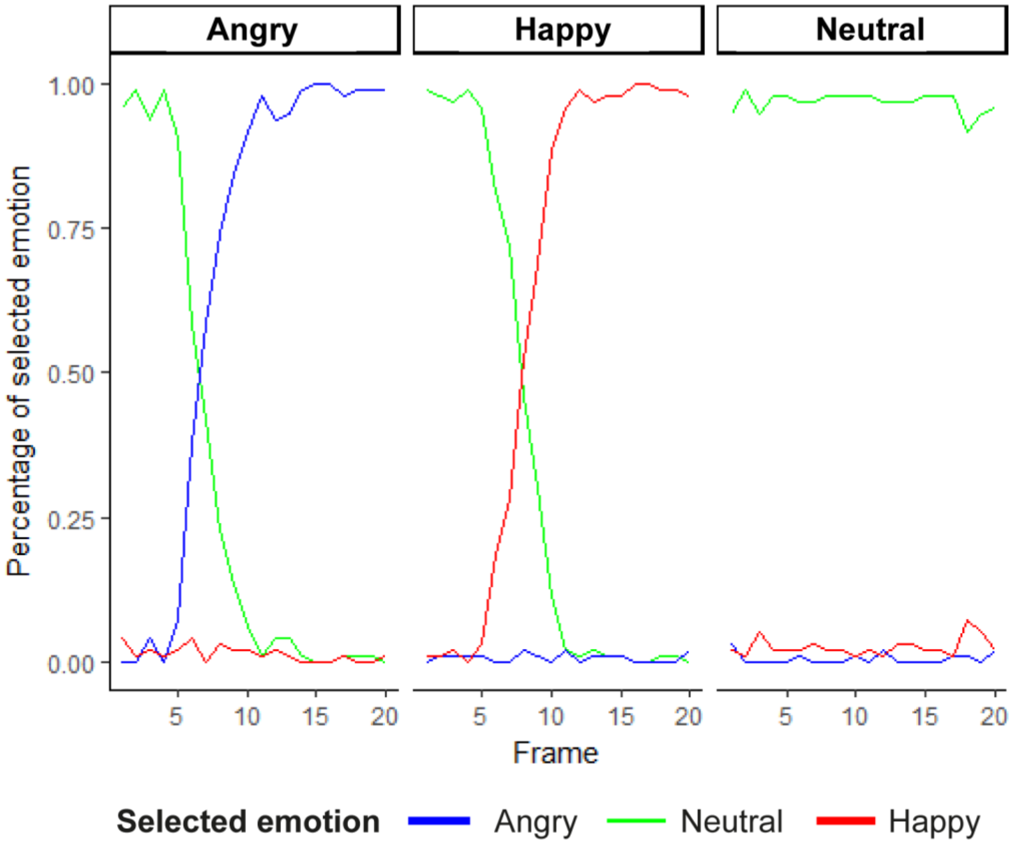

Figure S3: Proportion of selected emotions (line color) in the pre-test as a function of the true emotional expression of the video-clip (left, middle, right panel) and the frame number (x-axis).Frame number one corresponds to the first frame at which the transition from neutral to angry or happy was animated (neutral serves as a control with no transition).

Corrugator and Zygomaticus activation elicited by participants' display of the Initial Expression

Displaying an actual facial expression (i.e. the initial expression) resulted in a strong activation of the corresponding facial muscles. Display of an angry expression elicited activation mainly in the Corrugator muscle, and display of a happy expression elicited activation mainly in the Zygomaticus muscle. When facial expressions remained neutral, neither Corrugator nor Zygomaticus showed an increase in activation.

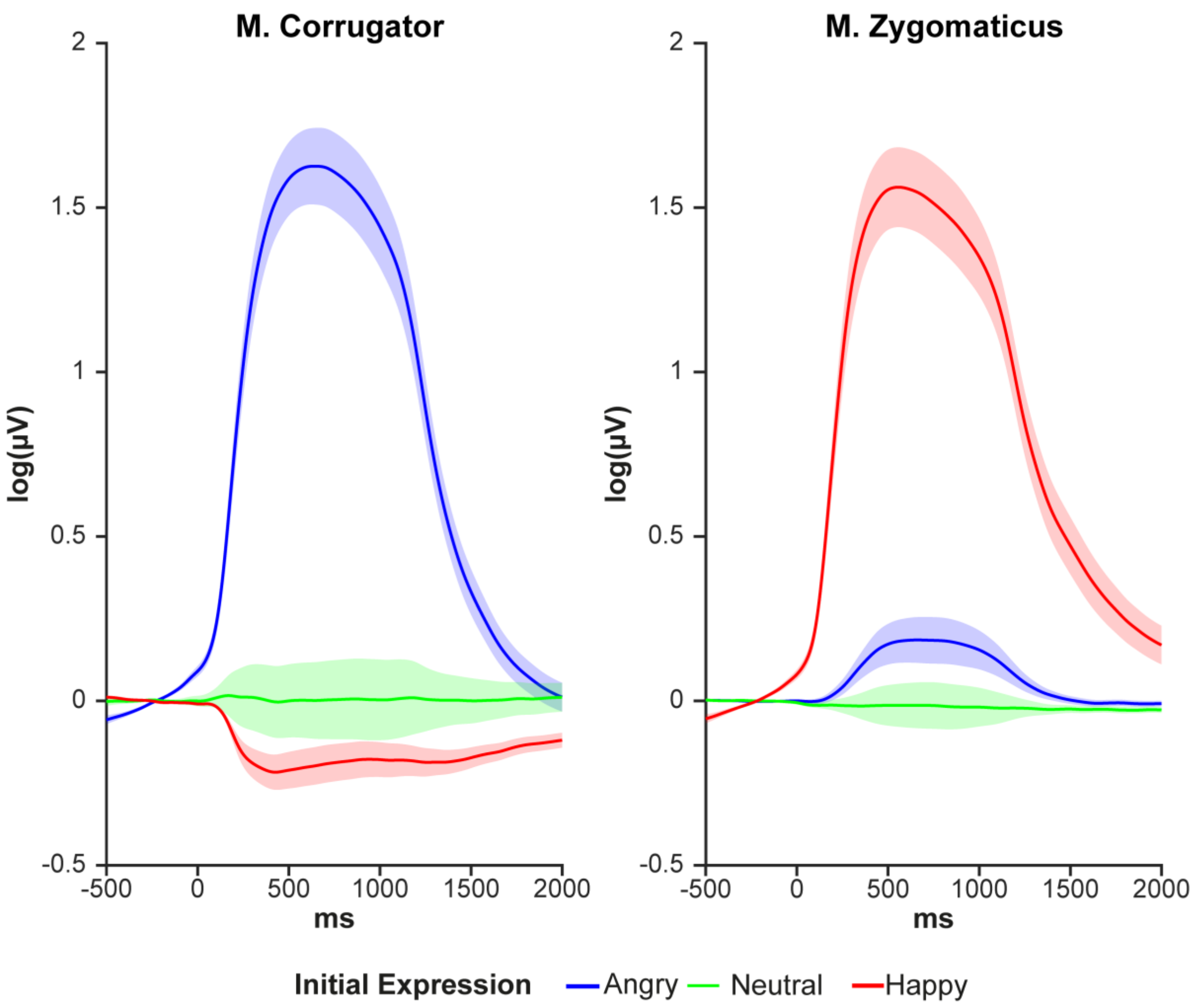

Figure S4: EMG activation in the Corrugator (left) and Zygomaticus (muscle) following the cue on which participants displayed the initial expression (angry, neutral, or happy). Shaded areas reflect the standard error of the mean.

ERP at Video Onset

ERP activation elicited by the onset of the facial stimulus (with a neutral expression) showed the typical pattern of positive and negative deflections at posterior temporo-occipital electrodes, i.e. the P1, N170, and EPN component.

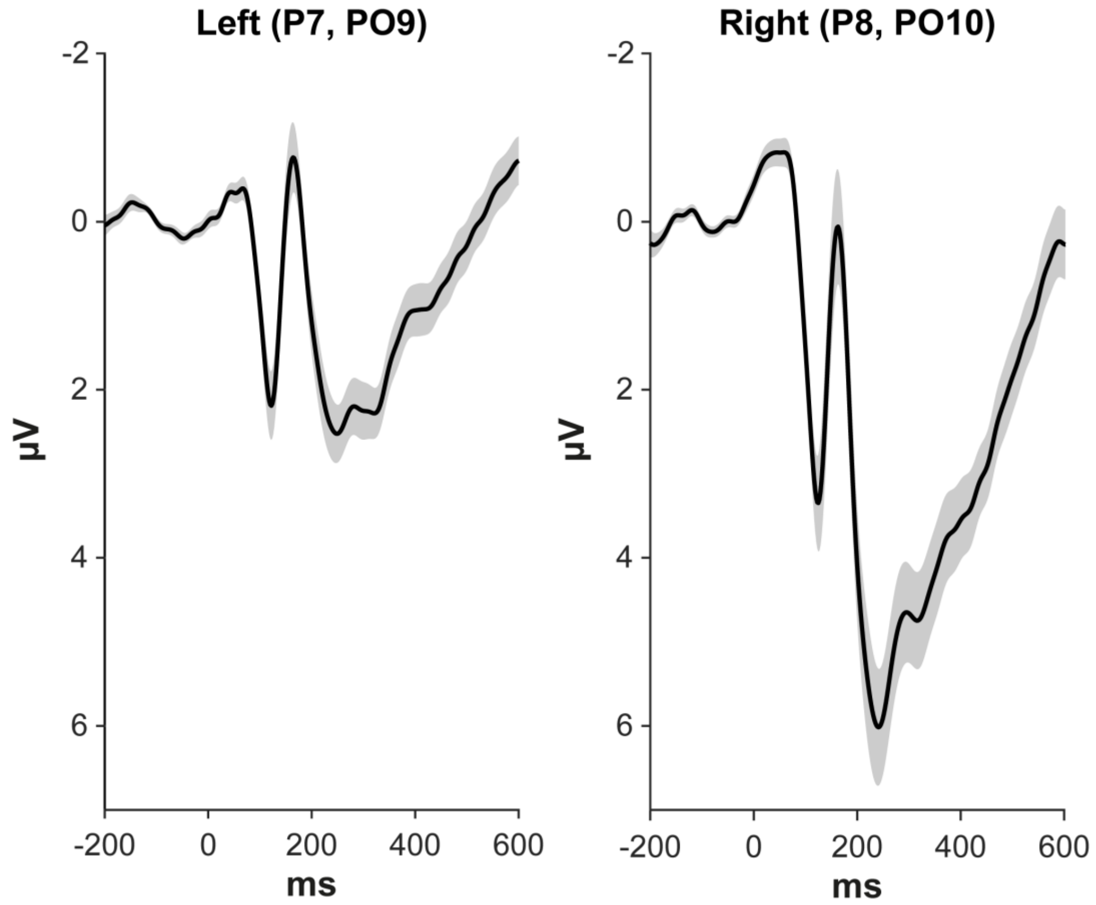

Figure S5: Grand average ERP time locked to the onset of the display of the virtual agent on the screen (with a neutral expression). Shaded areas reflect the standard error of the mean.

Single Condition ERP waveforms

The main analysis focused on difference waves between an emotional response expression (angry or happy) and the agent's neutral expression for each level of initial expression in order to account for lingering effects of muscle activation that was elicited by the display of an actual facial expression (see Figure S2). For completeness, ERP waveforms of all conditions are displayed here. Please note, that the slow drifts observed for initial angry and initial happy expression do result from the muscle activation and are eliminated by calculating the difference waves (see Figures 4 and 5). The graphs also show that the neutral expression of the agent does not result in particular ERP components.

EPN

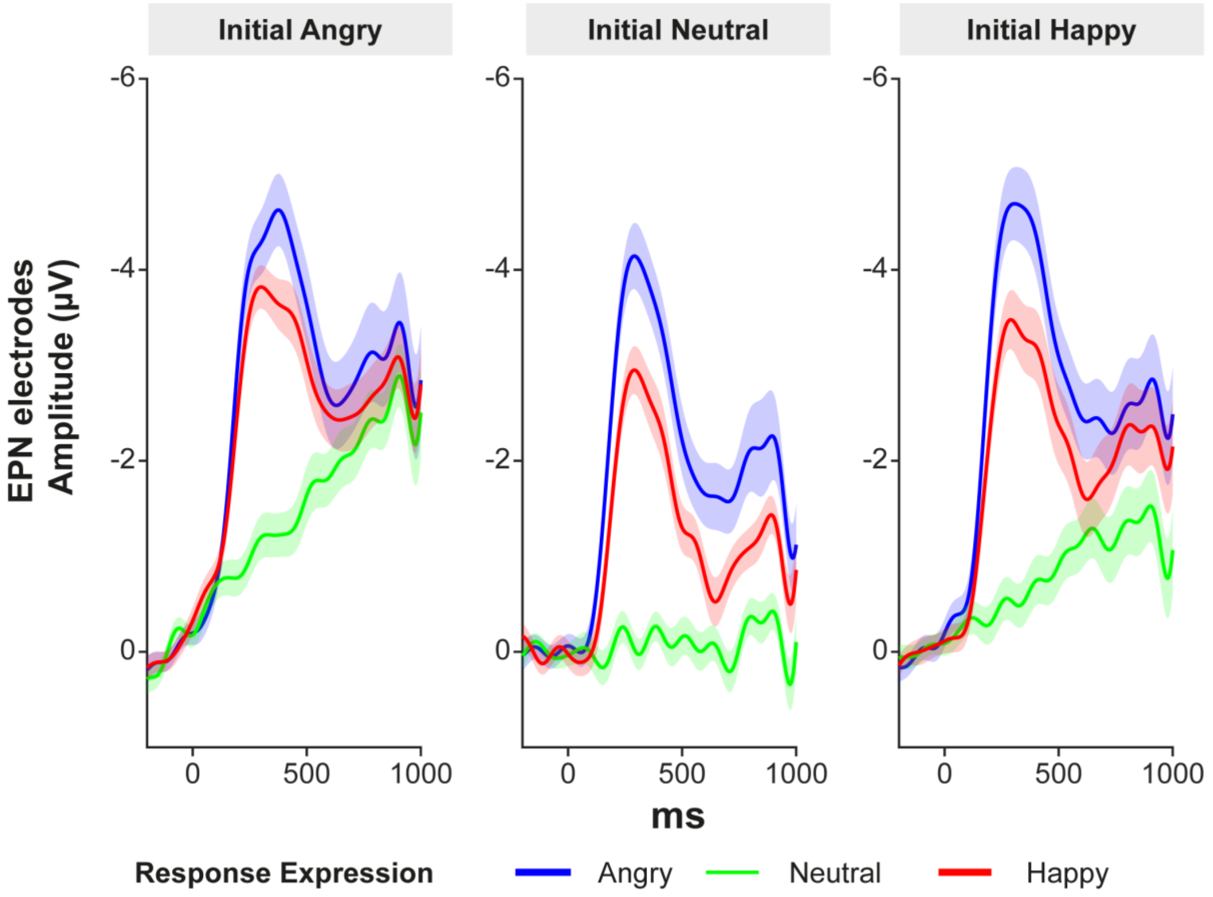

Figure S6: ERPs elicited by the agent's facial expression as a function of the initial emotional expression of the participant (left = angry, middle = neutral, right = happy)

and the response expression of the agent (angry = blue line, neutral = green line happy = red line) averaged across temporo-occipital electrode (PO7, PO8, P7, P8, O1, Oz, O2). Shaded areas reflect the standard error of the mean.

LPP

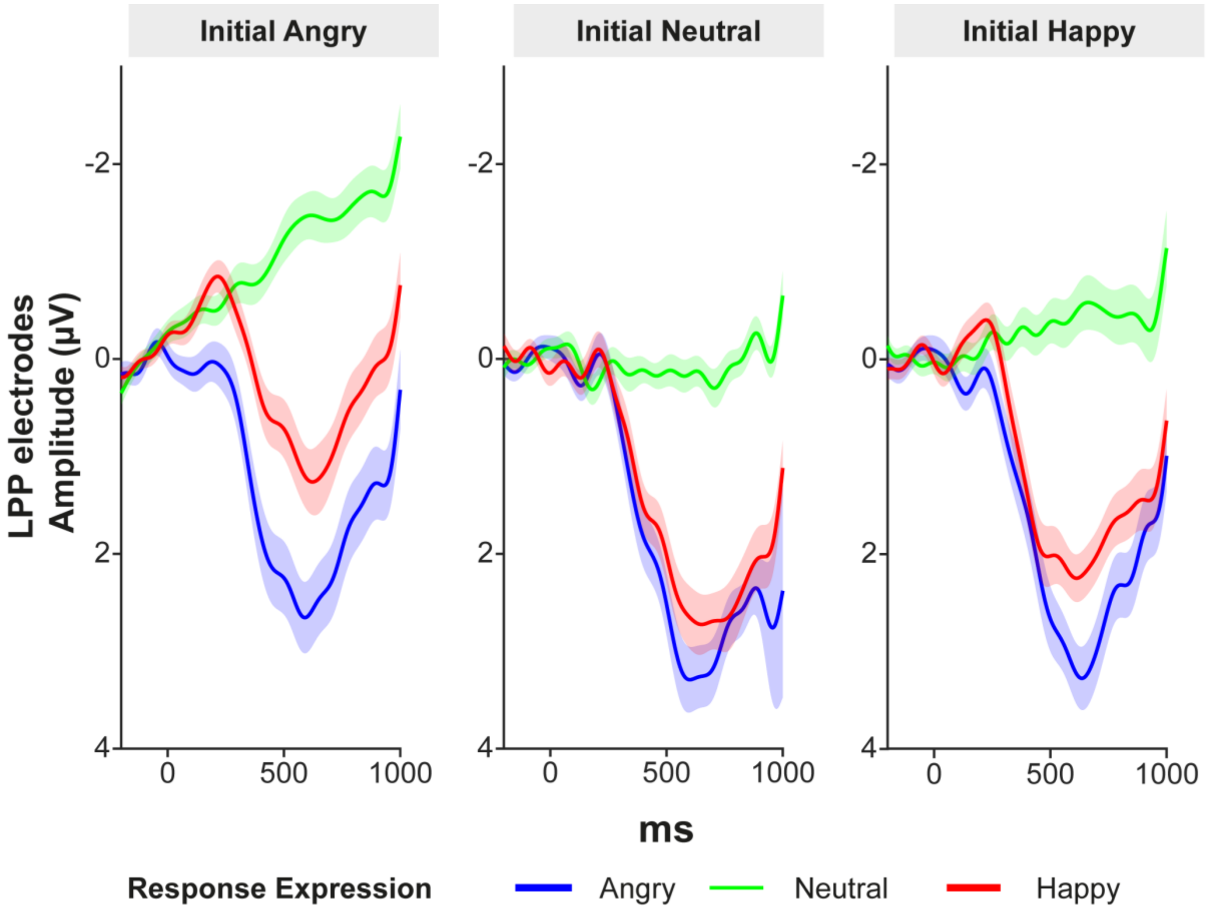

Figure S7: *ERPs elicited by the agent's facial expression as a function of the initial emotional expression of the participant (left = angry, middle = neutral, right = happy) and the response expression of the agent (angry = blue line, neutral = green line, happy = red line) averaged across centro-parietal electrode (CP1, CP2, Pz, P3, P4). Shaded areas reflect the standard error of the mean.*

Data-driven cluster-based permutation test

The main analysis of the EPN and LPP component was based on previously reported findings on ERP effects of facial emotional expressions. However, unlike most previous studies, the current paradigm presented the virtual agents for several seconds on the screen before the agent's facial expression changed dynamically from a neutral to an emotional expression. As a consequence, the typical N170 component for facial stimuli (Schindler et al., 2023) was not clearly identified following the emotional facial expression but only at the onset of the video clip when the agent first appeared (see Figure S3). Differences in paradigms may therefore reduce the validity of ROI and time window based analyses. Therefore, an additional data-driven analysis of the ERPs was conducted by entering all channels and timepoints in a cluster-based permutation test (Oostenveld et al., 2011). Clusters were defined on basis of channel and consecutive time points. Differences between angry and happy response expressions for each level of the initial expression were entered into a F-test for dependent samples to investigate interaction effects. In case of a significant effect, follow-up analyses were conducted by comparing response expressions within an initial expression condition using dependent t-tests. Monte Carlo simulations allowed to correct for multiple comparisons (N = 1000 randomizations, maximum of sum of F or t-values as cluster statistic).

Analyses of the interaction effect between Initial Expression and Response Expression were conducted by comparing the relative difference of angry and happy response expression between angry, neutral, and happy initial expressions using a univariate F-test. A significant interaction effect was observed in a centro-parietal cluster in a time window from 332 to 500 ms, cluster statistic = 1810.31, p = .021 (Figure S7 top). Follow-up analyses in that cluster for each initial expression showed that angry response expression elicited a greater positivity than happy response expression when

following an angry initial expression, cluster statistic = 4.98, $p < .001$, or a happy initial expression, cluster statistic = 2.22, $p = .008$, but not when following a neutral initial expression, cluster statistic = 1.98, $p > .05$.

In addition, analysis of the main effect of Response Expression using dependent t-tests revealed two significant clusters (Figure S7 bottom). A negative cluster (angry response expression being more negative than happy response expression) was observed over temporo-occipital electrodes in time window from 106 to 780 ms, cluster statistic = - 775.83, $p = .003$. In addition, a positive cluster (angry response expressions being more positive than happy response expressions) with a central distribution was observed in a sustained time window from 94 to 1 000 ms, cluster statistic = 1272.14, $p < .001$.

Finally, a cluster-based permutation test was conducted to test for the main effect of Initial Expression but no significant clusters were observed.

To conclude, results of the data-driven cluster-based permutation tests support the findings of the ROI-based analysis as they demonstrate a significant interaction effect in centro-parietal electrodes in a late time window which has been associated with the LPP component. Here, observation of an agent’s angry compared to a happy facial expression elicited a greater positivity, but only when participants had initiated the social exchange with an emotional but not neutral expression.

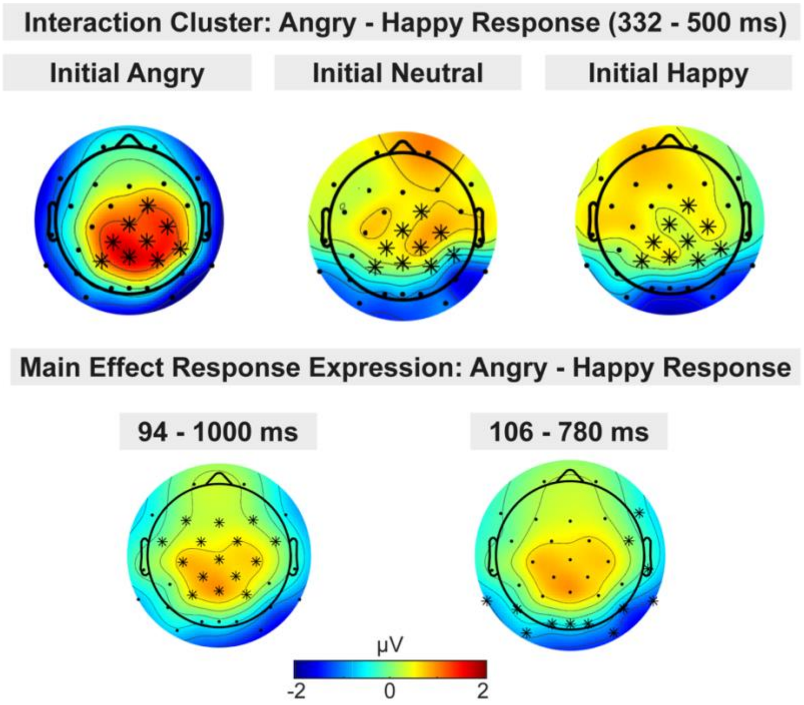

Figure S8: Results from Cluster-based permutation test. Top row shows topographical distribution of the effect of response expression (angry – happy) for each initial expression of 332 – 500 ms where a significant interaction effect was observed. Bottom: Positive and negative cluster related to the main effect of response expression (angry-happy). Electrodes included in the respectiveclusters are highlighted with asterisks.

Correlation between Ratings, EMG, and ERP

Table S1

Correlations between Valence, Arousal, Corrugator activity, Zygomaticus activity, EPN amplitude, LPP amplitude with confidence intervals.

Variable	Valence	Arousal	Corrugator	Zygomaticus	EPN
Valence					
Arousal	.05				
	[-.08, .17]				
Corrugator	-.15	.12			
	[-.28, -.02]	[-.02, .24]			
Zygomaticus	.25*	.02	-.12		
	[.12, .37]	[-.11, .15]	[-.25, .01]		
EPN	.18	-.09	-.11	.03	
	[.04, .30]	[-.22, .04]	[-.24, .02]	[-.10, .16]	
LPP	-.06	.04	.21*	.10	-.03
	[-.19, .07]	[-.10, .17]	[.09, .34]	[-.03, .23]	[-.16, .10]

Note. Values in square brackets indicate the 95% confidence interval for each

correlation. * indicates $p < .05$ after correction for multiple comparisons using Holm method
